# The combination of decitabine with multi-omics confirms the regulatory pattern of the correlation between DNA methylation of the CACNA1C gene and atrial fibrillation

**DOI:** 10.3389/fphar.2024.1497977

**Published:** 2024-12-13

**Authors:** Yuling Yang, Qijun Li, Xiaoning Liu, Caixia Shao, Heng Yang, Siquan Niu, Hong Peng, Xiangguang Meng

**Affiliations:** ^1^ Department of Pharmacy, Zhengzhou No. 7 People’s Hospital, Zhengzhou, Henan, China; ^2^ Department of Dermatology, Puyang Oilfield General Hospital, Puyang, Henan, China; ^3^ Medical School, Huanghe Science and Technology College, Zhengzhou, Henan, China; ^4^ Department of Surgery, Zhengzhou No. 7 People’s Hospital, Zhengzhou, Henan, China; ^5^ Department of Cardiac Surgery, Zhengzhou No. 7 People’s Hospital, Zhengzhou, Henan, China; ^6^ Department of Cardiology, Zhengzhou No. 7 People’s Hospital, Zhengzhou, Henan, China

**Keywords:** DNA methylation, calcium voltage-gated channel subunit alpha1 C, decitabine, Pyro-sequencing, atrial fibrillation

## Abstract

**Background:**

Studies have shown that DNA methylation of the CACNA1C gene is involved in the pathogenesis of various diseases and the mechanism of drug action. However, its relationship with atrial fibrillation (AF) remains largely unexplored.

**Objective:**

To investigate the association between DNA methylation of the CACNA1C gene and AF by combining decitabine (5-Aza-2′-deoxycytidine, AZA) treatment with multi-omics analysis.

**Methods:**

HepG2 cells were treated with AZA to observe the expression of the CACNA1C gene, which was further validated using gene expression microarrays. Pyrosequencing was employed to validate differentially methylated sites of the CACNA1C gene observed in DNA methylation microarrays. A custom DNA methylation dataset based on the MSigDB database was combined with ChIP-sequencing and RNA-sequencing data to explore the regulatory patterns of DNA methylation of the CACNA1C gene.

**Results:**

Treatment of HepG2 cells with three different concentrations of AZA (2.5 µM, 5.0 µM, and 10.0 µM) resulted in 1.6, 2.5, and 2.9-fold increases in the mRNA expression of the CACNA1C gene, respectively, compared to the DMSO group, with statistical significance at the highest concentration group (*p* < 0.05). Similarly, AZA treatment of T47D cells showed upregulated mRNA expression of the CACNA1C gene in the gene expression microarray results (adj *P* < 0.05). DNA methylation microarray analysis revealed that methylation of a CpG site in intron 30 of the CACNA1C gene may be associated with AF (adj *P* < 0.05). Pyrosequencing of this site and its adjacent two CpG sites demonstrated significant differences in DNA methylation levels between AF and sinus rhythm groups (*p* < 0.05). Subsequent multivariate logistic regression models confirmed that the DNA methylation degree of these three sites and their average was associated with AF (*p* < 0.05). Additionally, the UCSC browser combined with ChIP-sequencing revealed that the aforementioned region was enriched in enhancer markers H3K27ac and H3K4me1. Differential expression and pathway analysis of RNA-sequencing data ultimately identified ATF7IP and KAT2B genes as potential regulators of the CACNA1C gene.

**Conclusion:**

The DNA methylation levels at three CpG sites in intron 30 of the CACNA1C gene are associated with AF status, and potentially regulated by ATF7IP and KAT2B.

## 1 Introduction

Atrial Fibrillation (AF) is a disease that seriously affects public health, significantly impacting patients’ quality of life and prognosis. Although our understanding of AF’s pathogenic mechanisms has deepened in recent years, many key questions remain unanswered, particularly regarding the epigenetic regulation of CACNA1C gene DNA methylation, which is still in its early stages of research. Previous studies have shown that changes in CACNA1C gene function are associated with various physiological states and diseases (Lu et al., 2010; Assum et al., 2022; Desantis et al., 2023; Donniacuo et al., 2023; Andersen et al., 2021). Although limited current research has demonstrated that DNA methylation in the promoter region of this gene shows distinct tissue specificity in rats (Zhao et al., 2022), and in humans, CACNA1C gene DNA methylation modifications have been linked to psychiatric disorders (Penner-Goeke and Binder, 2019). However, there are few reports on whether changes in this gene’s DNA methylation are associated with AF. Therefore, understanding the relationship between this gene and AF from the perspective of DNA methylation will not only aid in disease diagnosis but also provide potential therapeutic targets for related drug development.

Decitabine (5-Aza-2′-deoxycytidine, AZA) is a DNA methyltransferase inhibitor that can cause DNA demethylation, thereby activating gene expression. It is primarily used in cancer treatment clinically (Dhillon, 2021). Research has shown that AZA treatment of different cells can determine whether target genes are related to DNA methylation (Habano et al., 2011). In our previous work, we treated HepG2 cells with AZA and found that the CYP3A4 gene was affected by DNA methylation (Wang et al., 2021). In this study, we similarly treated HepG2 cells with AZA to observe the effects of DNA methylation on CACNA1C gene expression.

Using original microarray and sequencing data from the Gene Expression Omnibus (GEO) database, many disease-related epigenetic markers can be identified (Palou-Marquez et al., 2021). The omics datasets used in this study are all from this database, including expression profiles, DNA methylation, ChIP-sequencing, and RNA-sequencing data.

In summary, based on the fact that AZA intervention reversed CACNA1C gene expression in HepG2 and T47D cell lines, combined with clinical samples and multi-omics statistical analysis, we identified that DNA methylation at three CpG sites in intron 30 of the CACNA1C gene is associated with AF status, and potentially regulated by ATF7IP and KAT2B. We hope to provide new perspectives and clues for epigenetic research in AF while also providing scientific evidence for the development of AF prevention and treatment strategies.

## 2 Materials and methods

### 2.1 Cell culture and processing

The human hepatocellular carcinoma cell line HepG2 was provided by the Shanghai Cell Bank, and the MycoEasy Rapid *Mycoplasma* Test Kit for *mycoplasma* contamination was provided by Beijing Cellapybio. The experimental process was conducted according to the manufacturer’s instructions. HepG2 was cultured in high-glucose medium containing 10% fetal bovine serum and 1% penicillin streptomycin mixture in DMEM at 37°C with a CO_2_ concentration of 5.0%. After treatment with methyltransferase inhibitors AZA 2.5, 5.0, and 10.0 μM from Sigma, St. Louis, MO, United States, and DMSO 0.1% v/v from Solarbio Technology, Beijing, China for 96 h, real-time fluorescence quantitative PCR (RT-qPCR) was performed.

### 2.2 Real-time fluorescence quantitative PCR

The expression level of CACNA1C mRNA in HepG2 cells before and after AZA treatment was detected using RT-qPCR. The SYBR GREEN method was used, with GAPDH as the internal reference gene, and the StepOnePlus™ system (applied Biosystems, Foster City, CA, United States) was used for fluorescence signal capture. The reaction system volume was 20 μL, and the reaction temperature conditions were 95°C for 120 s, 95°C for 15 s, and 60°C for 60 s, for a total of 40 cycles. The relative gene expression level was calculated using 2^−ΔΔCt^. Statistical analysis was performed using Student’s t-test. Data are expressed as mean ± standard deviation. The experiment was repeated 3 times.

### 2.3 Bioinformatics analysis

The gene expression microarray dataset GSE85536, sourced from AZA-treated human breast ductal carcinoma cell line T47D, was downloaded from the GEO database (Ariazi et al., 2017). Sequential analyses including quality control, normalization, filtering, annotation, and differential expression were performed on the raw data to verify whether the expression of CACNA1C gene mRNA was affected by AZA. The fold change threshold was set at |log2(FC)| > 1.58, with an adjusted *P*-value (adj*P*) < 0.05.

The DNA methylation microarray dataset GSE62727 related to AF was downloaded (Zhao et al., 2017), and potential DNA methylation sites associated with the CACNA1C gene were identified following the analytical workflow published by Jovana et al. (Maksimovic et al., 2016). Given the close relationship between DNA methylation and histone modifications, histone modification signatures were investigated within the regions of identified DNA methylation sites using seven built-in UCSC Browser datasets and their corresponding 13 datasets. These findings were then validated with ChIP-sequencing data from the GEO dataset GSE78113 (Platt et al., 2016) and two RNA-sequencing datasets (GSE153557 and GSE85353). Furthermore, a SE prediction analysis was conducted using a sample from GSE78113 through the ROSE software (Loven et al., 2013; Whyte et al., 2013), potentially providing valuable insights for future mechanistic studies.

The AF-related RNA-sequencing dataset GSE138255 was downloaded (Alvarez-Franco et al., 2021), and differentially expressed genes (DEGs) from the RNA-sequencing data were identified based on the optimized workflow published by Sahraeian et al. (2017). In this study, as our focus is on genes related to DNA methylation, we have customized a gene set named EpiGeneSet, which encompasses genes associated with DNA methylation, histone methylation, and histone acetylation, for subsequent data mining. The inclusion of the latter two is based on the fact that histone methylation and acetylation interact with DNA methylation. This custom gene set was selected from the C5 collection of the MSigDB database (c5. all.v2023.2. Hs.symbols.gmt). The GSVA package was utilized to observe the enrichment of pathways related to DNA methylation, histone methylation, and acetylation, and to explore the co-expression patterns with the CACNA1C gene. The fold change threshold for both differential gene expression analysis and GSVA analysis was set at |log2(FC)| > 0.58, with adj*P* < 0.05. The reference genome used was Ovis_aries.Oar_v3.1.99.

### 2.4 Patient information

Fifteen patients with permanent AF and 24 patients with sinus rhythm (SR) from Zhengzhou No. 7 People’s Hospital were included in this study. All subjects were hypertensive and had undergone cardiac surgery due to coronary heart disease or mitral valve disease, with an AF status lasting for more than 3 months. Other diseases such as lung disease, diabetes, hyperthyroidism, rheumatism, autoimmune diseases, congenital heart disease, heart failure, and myocardial bridging were excluded. The clinical data of all patients are presented in Table 1. The left atrial appendage tissues (LAA) from the patients were rapidly frozen in liquid nitrogen and then stored in an ultra-low temperature freezer at −80°C for further processing. All procedures complied with the Helsinki Declaration, and written informed consent was obtained from all participants. This study was approved by the Ethics Committee of Zhengzhou No. 7 People’s Hospital (Approval No. 20190804).

**TABLE 1:** Data characteristics of AF and SR groups.

Factors		AF (n = 15)	SR (n = 24)	χ^2^/W	*P*-value	95% CI
Sex	Male	6	16	2.67	0.10	
	Female	9	8			
Smoke	Yes	5	12	1.04	0.31	
	No	10	12			
Drink	Yes	4	11	1.43	0.23	
	No	11	13			
Age		63.00 (55.50, 66.00)	54.50 (51.00, 70.50)	214.50	0.33	−4.00, 11.00
Meth. of Site_1		16.13 (15.34, 17.54)	19.38 (17.17, 20.85)	85	0.0064*	−4.21, −0.72
Meth. of Site_2		24.57 (23.81, 26.14)	27.84 (24.93, 29.50)	112	0.051	−4.86, 0.040
Meth. of Site_3		35.16 (34.00, 37.84)	41.05 (36.27, 43.13)	90	0.0098*	−7.32, −1.21
Meth. of Site_M		25.23 (24.45, 27.04)	29.39 (25.87, 30.74)	104	0.029*	−5.00, −0.32

*: *P* < 0.05; AF, atrial fibrillation; Meth., methylation; SR, sinus rhythm.

### 2.5 Pyrosequencing for DNA methylation

After tissue sample extraction, 0.5–1 μg of DNA was subjected to C-U base conversion using the Qiagen epitect bisulfite Kit, followed by PCR amplification. Sequencing primers were designed using PyroMark Assay Design 2.0 software and synthesized by Life Technology. The products were then subjected to pyrosequencing using the PyroMark Q48 sequencing system (Qiagen, Hilden, Germany). The methylation status of each CpG site was automatically analyzed using Pyro Q-CpG software. The 50 μL reaction mixture contained 2 μL of DNA template, 10 μL of 5 * KAPA GC buffer, 1 μL of 10 mM dNTP, 1 μL of 50 p.m./μL forward or reverse primer, and 0.2 μL of 5 U/μL Taq. The reaction conditions were as follows: 95°C for 180 s, followed by 40 cycles of 94°C for 30 s and 56°C for 30 s.

### 2.6 Statistical analysis

**FIGURE 1:**
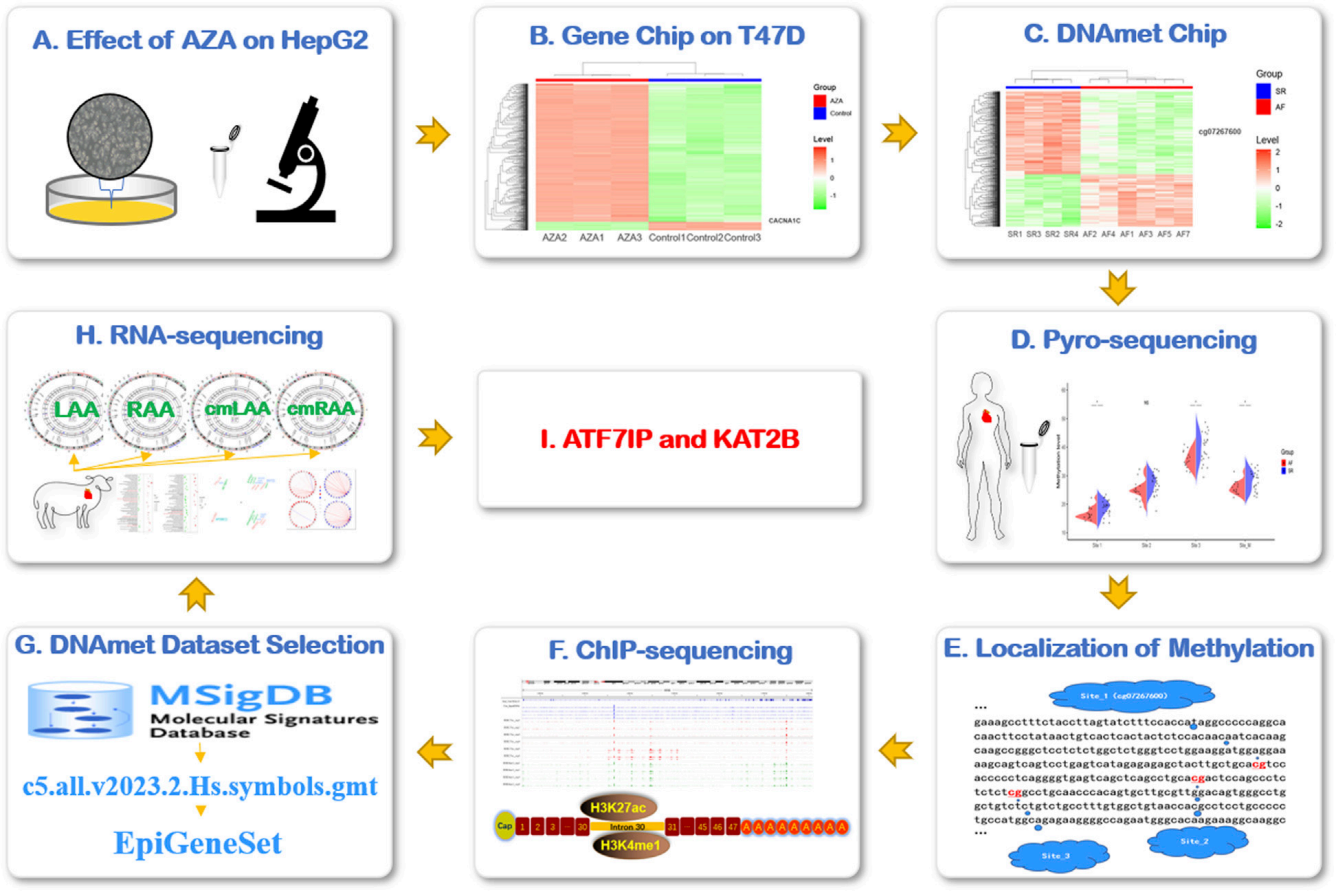
Research flowchart. **(A)** AZA interfered in HepG2 cells to observe the expression of CACNA1C gene. **(B)** The expression of CACNA1C gene was verified by gene expression profiling chip data (AZA-treated human breast ductal carcinoma cell line T47D). **(C)** To explore the possible AF-related DNA methylation sites of CACNA1C gene in DNA methylation chip dataset. **(D)** The degree of DNA methylation sites for CACNA1C gene was verified in the LAA of AF patients by pyrosequencing. **(E)** The locations of the three DNA methylation sites of interest. **(F)** The above three CpG locus regions were found to be strong concentrations of enhancer indicator signals H3K4me1 and H3K27ac in the ChIP-sequencing data set. **(G)** The C5 dataset of MSigDB database was used to customize the DNA methylation-related gene set EpiGeneSet for subsequent analysis. **(H)** The EpiGeneSet dataset combine RNA-sequencing data to explore network co-expression patterns with CACNA1C genes. **(I)** ATF7IP and KAT2B may be involved in the regulation of DNA methylation of CACNA1C gene. AZA: Decitabine, cmLAA: left atrial appendage for cardiac muscle cell; cmRAA: right atrial appendage for cardiac muscle cell; DNAmet: DNA methylation, LAA: left atrial appendage for cardiac muscle tissue; RAA: right atrial appendage for cardiac muscle tissue.

The overall analysis workflow of this study is shown in Figure 1, and all primers used are listed in Table 2. All data analyses were performed using R version 4.2.1 and its related packages (R Core Team, 2013). Chi-square tests were used for categorical variables (Sex, Smoke and Drink), and the Wilcoxon test was used for continuous variables (Age). Multivariate logistic regression models were employed to investigate the association between DNA methylation levels of CpGs and AF. A *P*-value less than 0.05 was considered statistically significant, indicating a significant association between the two variables.

**TABLE 2:** Primer list.

Symbol	Sequence	Length (bp)	Organism	Type
CACNA1C	GCCCCGAAACATGAGCAT	217	HepG2	RT-qPCR
	GAA​AAT​CAC​CAG​CCA​GTA​GAA​GA			
GAPDH	AAC​AGC​GAC​ACC​CAC​TCC​TC	258	HepG2	
	GGA​GGG​GAG​ATT​CAG​TGT​GGT			
CACNA1C	AGG​TAG​ATA​GAG​ATA​GTT​AGG​TTT​ATT	201	Human	DNA Methylation
	^a^CAA​ACC​CAA​CTC​CTC​TCT​AAC​TCT​AAA​TCC			
	^b^TTATTGTTTAAAGTAAGTATTGTGG			

^a^5′-Biotin.

^b^Sequencing primer.

## 3 Results

### 3.1 AZA increases mRNA expression of the CACNA1C gene in HepG2 cells

**FIGURE 2:**
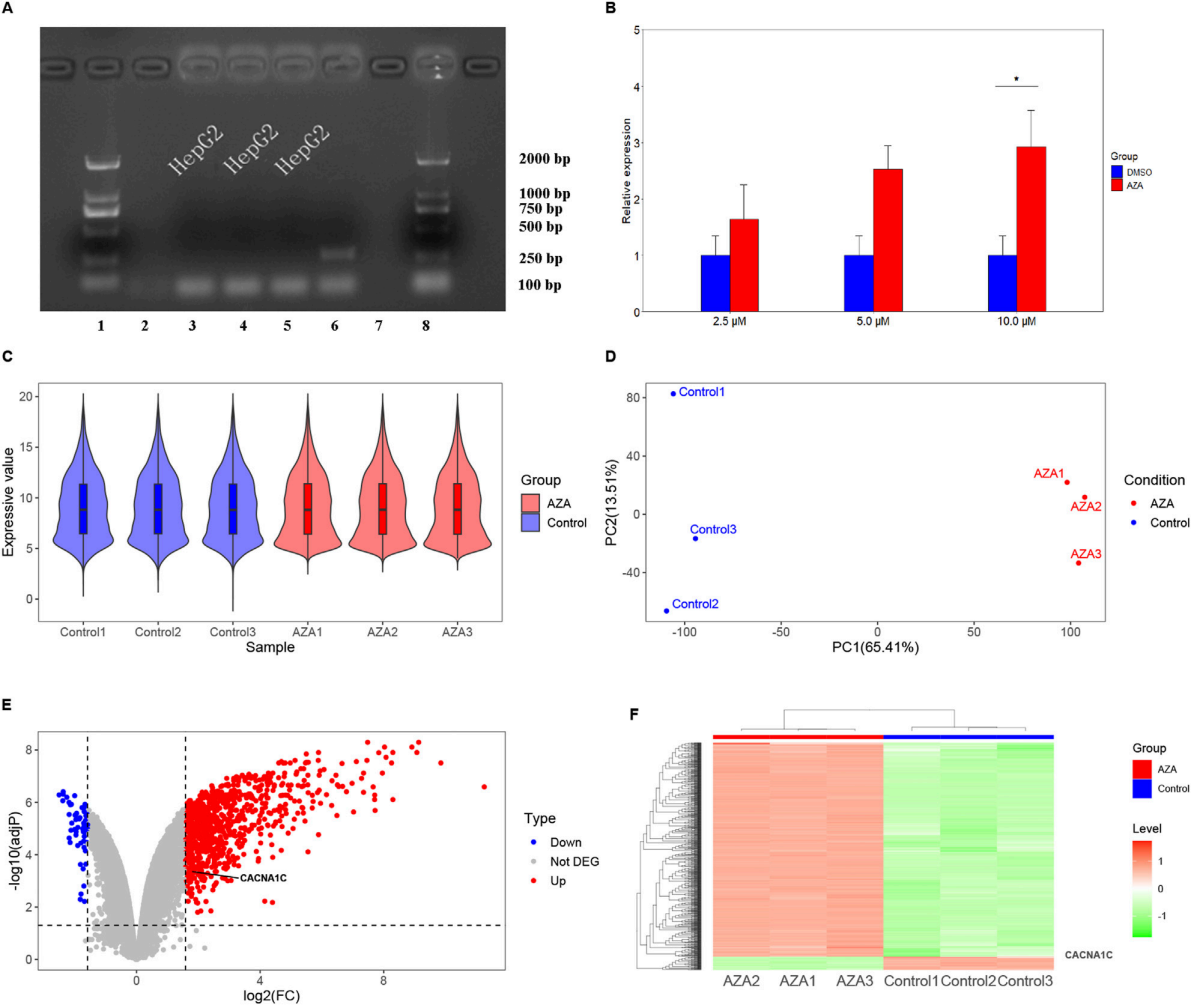
Observation of CACNA1C gene expression alteration in HepG2 and T47D cells induced by AZA using RT-qPCR methods and reanalysis of chip data from the GEO Database. **(A)** *Mycoplasma* detection was performed in HepG2 cells. On the *x*-axis, lanes 1 and eight represent the LD2000 DNA marker, lanes two and 7 represent the negative controls, lane 6 represents the positive control, and the remaining lanes three to five represent the HepG2 cells used for subsequent experiments. The *y*-axis indicates the specific positions of the DNA marker bands. **(B)** RT-qPCR results comparing three concentrations of AZA (2.5 µM, 5.0 µM, and 10.0 µM) treated HepG2 cells for 96 h with the control group (DMSO). **(C)** Violin plot showing the distribution of normalized data from all samples after hybridization of a genome expression profiling chip following AZA intervention in T47D cells. **(D)** Principal component analysis plot of the samples. **(E)** Volcano plot depicting the expression of the CACNA1C gene in T47D cells. **(F)** Heatmap showing the expression of the CACNA1C gene in T47D cells.

Initially, the HepG2 cells were tested for *mycoplasma* contamination, and the findings were negative, as shown in Figure 2A. Subsequently, the effect of AZA on the mRNA expression of the CACNA1C gene was evaluated by RT-qPCR, and the results revealed that treatment of HepG2 cells with three concentrations of AZA (2.5 µM, 5.0 µM, and 10.0 µM) for 96 h led to 1.6-, 2.5-, and 2.9-fold increases in the expression of CACNA1C mRNA, respectively, compared to the control group (DMSO). Although statistical significance was only observed at the highest concentration (*P* < 0.05, Figure 2B), the trend across the three different concentrations showed a gradual increase. These results indicate that AZA treatment of HepG2 cells can influence the mRNA expression of the CACNA1C gene.

### 3.2 AZA increases mRNA expression of the CACNA1C gene in T47D cells

After treating T47D estrogen-resistant cells with AZA and 0.1% ethanol, gene chip hybridization was performed with three samples in the AZA group and three samples in the control group. The obtained expression profile data were analyzed. The violin plot demonstrated a uniform distribution of the normalized samples in both groups (Figure 2C). Principal component analysis (PCA) revealed intra-group clustering among the samples, indicating similar intra-group distances (Figure 2D). The volcano plot presented the differences in gene expression between groups, with 871 upregulated genes and 55 downregulated genes, as shown in Figure 2E. Notably, CACNA1C was among the upregulated genes, with a fold change of 1.74 and an adj*P* value of 0.00043. The heatmap generated using expression signals also highlighted the location of the upregulated CACNA1C gene (Figure 2F). This gene chip confirmed that AZA can also increase the mRNA expression of the CACNA1C gene in other cell lines.

### 3.3 DNA methylation of one CpG site are potentially associated with AF in intron 30 of the CACNA1C gene

**FIGURE 3:**
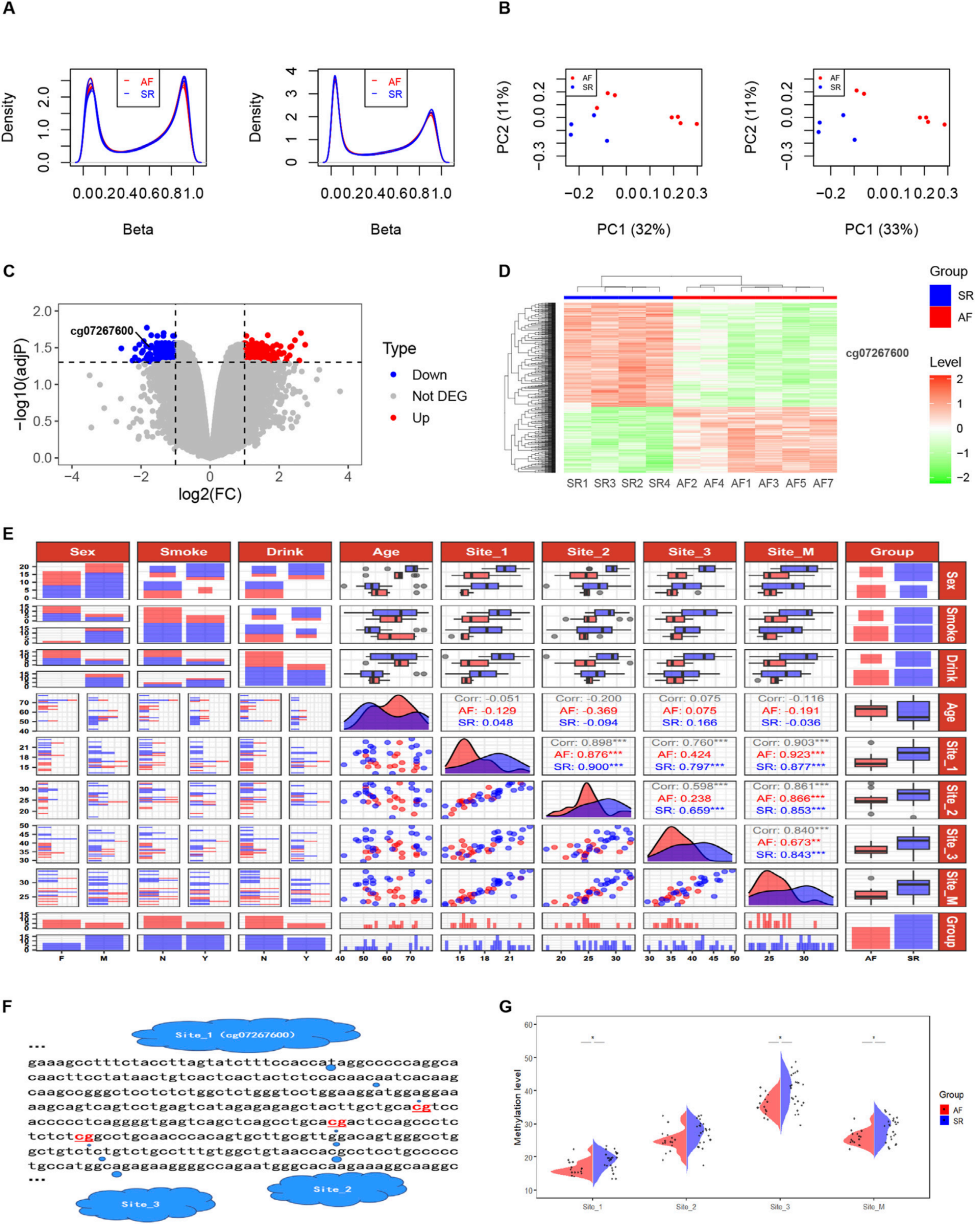
DNA methylation levels at three CpG sites in intron 30 of the CACNA1C gene correlate with AF. **(A)** Comparison of Beta values from DNA methylation chip data before and after normalization. **(B)** MDS analysis comparison before and after removal of sample AF6. **(C)** Volcano plot displaying differential enrichment of the CACNA1C gene DNA methylation probe cg07267600 between groups. **(D)** Heatmap showing differential enrichment of the CACNA1C gene DNA methylation probe cg07267600 between groups. **(E)** Data distribution and correlation diagram for clinical factors, Site_1, Site_2, Site_3, and Site_M. Categorical variables such as sex, smoke, and drink are presented using bar charts or stacked bar charts. Continuous variables including age and methylation levels at sequencing sites are represented by box plots and density plots, while correlations between groups are depicted by scatter plots. **(F)** Specific locations of the three CpG sites in intron 30 of the CACNA1C gene. **(G)** Violin plots illustrating the differences of DNA methylation levels at the three CpG sites in intron 30 of the CACNA1C gene between the AF and SR groups. * indicates statistically significant difference; Site_M represents the mean percentage of methylation levels at the three sites.

Quality control was performed on AF-related DNA methylation chip data consisting of 11 samples (AF group: n = 7 and SR group: n = 4). Figure 3A demonstrates that after normalization, the distribution of DNA methylation samples becomes more uniform. The multidimensional scaling (MDS) analysis was carried out, following the probes filtered out (including with low-quality probes, located on X/Y chromosomes, with mutations in CpG sites, and where a single CpG site aligns to multiple locations in the genome). An intra-group clustering tendency was observed for the two groups, particularly after the exclusion of sample AF6 (Figure 3B). Differential analysis of DNA methylation CpG sites between the cleaned sample groups using the Limma package revealed 346 upregulated and 546 downregulated sites, as detailed in Supplementary Table S1. Notably, cg07267600, a CpG probe located within intron 30 of the CACNA1C gene, exhibited a significant difference between groups (log2(FC) = −1.73, adj*P* = 0.032). The volcano plot (Figure 3C) and clustering heatmap (Figure 3D) illustrate lower expression levels of this probe in the AF group. Further analysis revealed that this site is of the OpenSea type and is adjacent to two other CpG sites downstream (Figure 3F), suggesting the need for further validation of the association between DNA methylation at these three sites and AF.

### 3.4 DNA methylation of three CpG sites associated with AF were identified in intron 30 of the CACNA1C gene

Pyrosequencing of DNA methylation in LAA tissues from AF and SR patients was performed, and a trend plot depicting the distribution of sequencing data and clinical variables was generated (Figure 3E). Potential collinearity was observed among Site_1, Site_2, Site_3, and Site_M (collinearity is commonly reported among neighboring CpG sites [31078719 2020 4.4]). This collinearity was confirmed using variance inflation factor (VIF) analysis from a logistic regression model, with all four variables having VIF values greater than 10 (Supplementary Table S2). Consequently, subsequent multivariable logistic regression analyses modeled individual CpG sites separately with covariates (Age, Sex, Drink, and Smoke). Wilcoxon tests revealed significant differences in DNA methylation levels between the AF and SR groups at Site_1, Site_3, and Site_M (mean of the three sites) (*P* < 0.05), while Site_2 showed non-significant but near-significant differences (Figure 3G). Results from four multivariable logistic regression models confirmed the association of Site_1, Site_2, Site_3, and Site_M with AF (*P* < 0.05), as presented in Table 3. In summary, these findings suggest that the DNA methylation degree of three CpG sites in intron 30 of the CACNA1C gene influences AF.

**TABLE 3:** Construction of logistic regression models for DNA methylation sites.

Variables	Method	Coef.	*P*-value	95% CI
Inter.	Model_Site_1	14.76	0.013*	4.20, 28.03
Age		−0.038	0.46	−0.15, 0.060
Sex		−2.37	0.10	−5.55, 0.34
Smoke		−0.74	0.48	−2.87, 1.32
Drink		−0.19	0.88	−2.62, 2.38
Site_1		−0.64	0.0052*	−1.18, −0.25
Inter.	Model_Site_2	9.94	0.065	−0.033, 21.57
Age		−0.037	0.45	−0.14, 0.057
Sex		−1.51	0.24	−4.36, 0.91
Smoke		−0.49	0.60	−2.34, 1.40
Drink		−0.17	0.88	−2.37, 2.25
Site_2		−0.27	0.032*	−0.55, −0.046
Inter.	Model_Site_3	12.15	0.029*	2.31, 24.81
Age		−0.00058	0.99	−0.11, 0.10
Sex		−1.44	0.26	−4.15, 1.11
Smoke		−0.21	0.83	−2.15, 1.81
Drink		−0.56	0.64	−2.94, 1.92
Site_3		−0.31	0.0075*	−0.57, −0.11
Inter.	Model_Site_M	12.97	0.026*	2.41, 25.79
Age		−0.033	0.51	−0.14, 0.063
Sex		−1.58	0.22	−4.33, 0.88
Smoke		−0.43	0.65	−2.33, 1.52
Drink		−0.31	0.79	−2.61, 2.13
Site_M		−0.38	0.011*	−0.72, −0.12

*: *P* < 0.05; Coef., coefficient; Inter., intercept.

### 3.5 The enrichment region of enhancer markers H3K27ac and H3K4me1 overlap with three CpG loci

**FIGURE 4:**
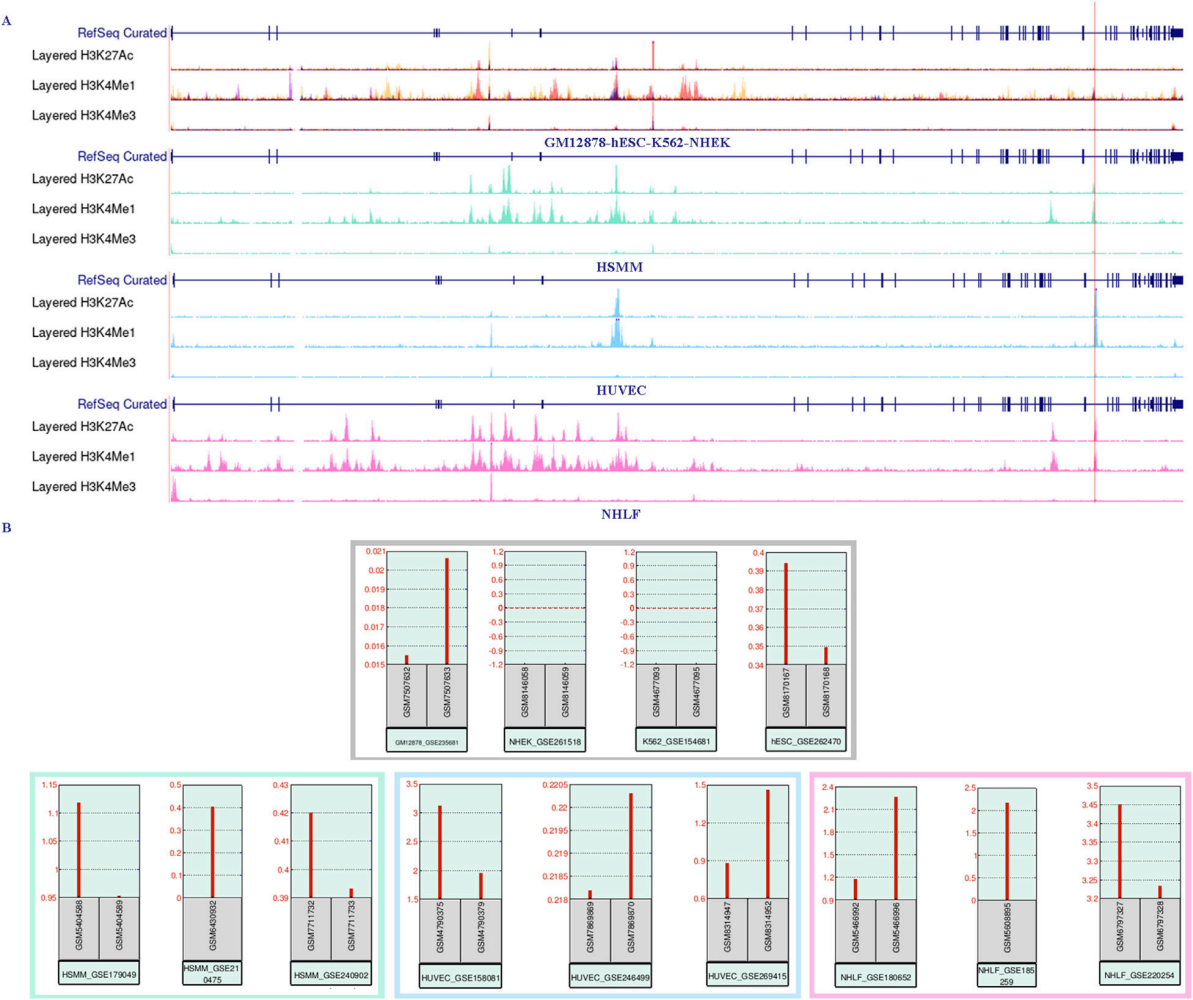
RNA-sequencing expression data shows a positive correlation with the enrichment of the enhancer marker H3K27ac near the three CpG sites in intron 30 of the CACNA1C gene. **(A)** The UCSC Genome Browser shows the enrichment of histone markers H3K27ac, H3K4me1, and H3K4me3 across seven different cell lines. The upper panel represents the lack of enrichment of the three histone markers in the GM12878, hESC, K562, and NHEK cell lines. The bottom three panels, in contrast, demonstrate strong enrichment of H3K27ac in the HSMM, HUVEC, and NHLF cell lines. The red vertical line marks the span of the three CpG sites. The ChIP-sequencing data was obtained from the UCSC Genome Browser. **(B)** CACNA1C gene expression levels observed from RNA-sequencing data in the same seven cell lines. The upper panel shows CACNA1C expression in GM12878, hESC, K562, and NHEK cell lines, while the lower panel presents expression data from HSMM, HUVEC, and NHLF cell lines. The RNA-sequencing data is sourced from the GEO database.

Using the UCSC Genome Browser, we examined three enhancer-enriched histone modification tracks (H3K27ac, H3K4me1, and H3K4me3) across seven cell lines (HSMM, HUVEC, NHLF, GM12878, K562, NHEK, and hESC). We observed a high enrichment of H3K27ac and H3K4me1 signals in the intron 30 region of the CACNA1C gene in the first three cell lines, which also overlapped with three CpG sites. This suggests that the region may function as a gene enhancer or super-enhancer element (see Figure 4A, bottom three panels). Next, we explored the expression of CACNA1C using RNA-sequencing data from these three cell lines (downloaded from GEO) via the geo2r tool. Results showed that CACNA1C expression (TPM > 0.1) was consistent across all nine datasets (three for each cell line), as illustrated in the bottom panels of Figure 4B. In contrast, no H3K27ac enrichment was found in the remaining four cell lines (see Figure 4A, top panels), where CACNA1C expression was either absent or minimal (TPM < 0.1), except in hESC (Figure 4B, top panels). This observation led us to hypothesize that this enhancer element could be involved in regulating CACNA1C expression. To further investigate, we identified a dataset from GEO (GSE78113) that includes ChIP-sequencing results for multiple enhancer markers (H3K27ac, H3K4me1, H3K4me3, and CTCF) in the Breast Cancer Cell Line-MCF-7, both under normal and hypoxic conditions.

**FIGURE 5:**
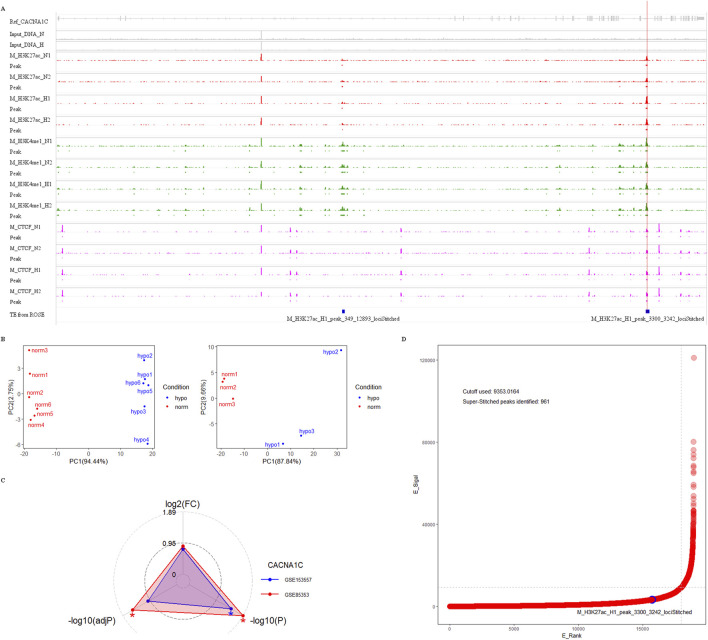
Genomic analysis of enhancer markers H3K27ac, H3K4me1, H3K4me3, and CTCF protein near three CpG sites in intron 30 of the CACNA1C gene. **(A)** Analysis of ChIP dataset GSE78113 using Integrative Genomics Viewer to observe the enrichment signals of the four markers near the three CpG sites. The dataset comes from MCF-7 cell lines under normal conditions (track names ending with N) and hypoxic conditions (track names ending with H). Four red tracks show H3K27ac enrichment signals, four green tracks show H3K4me1 enrichment signals, and four pink tracks show CTCF protein enrichment signals. Their corresponding enrichment peaks are shown in Peak tracks below each signal track, represented by rectangles filled with corresponding colors. Additionally, the top gray track shows the structure of the reference gene CACNA1C. The second and third tracks show enrichment signals from two Input DNAs, while the bottom blue track shows enhancer elements predicted using ROSE software. The letter M in track names represents the MCF-7 cell line. The red vertical line in the plot indicates the span of the three CpG sites. **(B)** Quality control of two RNA-sequencing datasets from MCF-7 cell lines. In the PCA analysis plots, red dots represent six normal samples, while blue dots represent hypoxic samples. The left dataset is from GEO GSE85358, and the right dataset is from GEO GSE153557. **(C)** Radar plot showing CACNA1C gene expression differences between normal and hypoxic groups in the above two RNA-sequencing datasets. The red triangle’s apex represents the fold change value of CACNA1C gene expression in GSE85358, the left base point represents the adj*P* value, and the right base point represents the *P*-value, while the blue triangle’s vertices represent the corresponding three values for GSE153557. **(D)** Distribution plot of TE/SE elements predicted by ROSE software in ChIP dataset sample M_H3K27ac_N1. The *x*-axis E_Rank represents the ranking of all enhancer signals, the *y*-axis E_Signal represents the difference between H3K27ac enrichment signal and Input DNA enrichment signal. The blue circle indicates the predicted TE in intron 30 of the CACNA1C gene, the horizontal dashed line represents the SE enrichment signal threshold: 9353.0164, and the vertical dashed line represents the SE rank threshold: 18014. TE: typical enhancer, SE: super-enhancer.

For this dataset, we used Bowtie2 + MACS2 for peak calling, and generated bigwig files using Deeptools, which were then imported into the IGV browser. As shown in Figure 5A, there are clear enrichment peaks for H3K27ac and H3K4me1 signals at three CpG sites in intron 30 of the CACNA1C gene, highlighted by red and green tracks. These peaks are more prominent in the hypoxia group compared to the normal group, suggesting that this change may be associated with increased gene expression. Since gene expression data was not available in this dataset, we used two other MCF-7 datasets (GSE85353 and GSE153557) to investigate expression differences between the normal and hypoxia groups. The PCA plots from the cleaned data of both datasets show clear group separation, with strong intra-group clustering (see Figure 5B). A radar plot revealed significantly higher CACNA1C expression in the hypoxia group in dataset GSE85353 (*P* < 0.05, adj*P* < 0.05), while GSE153557 also showed a *P* < 0.05 difference, though adj*P* was not significant. Both datasets demonstrated a high fold change between groups, approximately 0.8 (Figure 5C). Thus, we propose that the activation of this enhancer may positively regulate CACNA1C expression.

Additionally, the region is highly enriched with CTCF protein, which is a marker for super-enhancers, along with H3K27ac and H3K4me1 (Figure 5A, purple track). To investigate whether this enhancer could form a super-enhancer, we selected the M_H3K27ac_N1 sample and used the ROSE super-enhancer prediction tool to analyze the intron 30 region of the CACNA1C gene. The results indicate that while the position did not meet the super-enhancer threshold (9353.0164), it exhibited a strong TE signal with a score of 3,242 (Figure 5A, blue track and Figure 5D). The raw ROSE data is shown in Supplementary Table S3. These findings suggest that this region is a key regulatory element with potential pathophysiological and pharmacological significance, and further experiments are required to determine if it plays a role in the progression of AF and whether it can form a super-enhancer.

In summary, the enrichment of enhancer markers H3K27ac, H3K4me1, and CTCF at three CpG sites in intron 30 of the CACNA1C gene supports its role in gene expression. This also implies that changes in DNA methylation in this region may influence these histone modifications, potentially regulating CACNA1C expression. Furthermore, to further explore potential regulatory genes, we screened an EpiGeneSet (comprising 447 genes, see Supplementary Table S4) related to these epigenetic regulations from the C5 dataset of the MSigDB database for downstream analysis.

### 3.6 Epigenetic regulatory genes potentially related to the CACNA1C were identified based on RNA-sequencing

The RNA-sequencing dataset GSE138255 comprises four groups of sheep in different states: Transition group (self-sustaining AF without conversion to SR, lasting 13.75 ± 4.50 days), Chronic group (self-sustaining AF without conversion to SR, lasting 289.25 ± 50.29 days), Sham group (sham-treated, SR), and Control group (unoperated animals, SR). Due to the limited statistical power caused by the Control group containing only two samples, it was excluded (the latter two groups were merged in the original article). Heart sampling from sheep involved both tissue and cellular levels: tissues included LAA and right atrial appendage (RAA); cells included cardiomyocytes from LAA (cmLAA) and cardiomyocytes from RAA (cmRAA). All samples were processed through the HISAT2 + FeatureCounts + DESeq2 pipeline, yielding a total of 8 DEG sets, as detailed in Supplementary Table S5. In addition, the first four rows of Table 4 show the number of DEGs between each group (Transition-Sham or Chronic-Sham) for the eight datasets. The results indicate that in LAA, cmLAA, and cmRAA, the overall gene expression level in the Chronic group was significantly higher than in the Transition group, while in RAA, it was reduced, likely due to the tissue’s heterogeneity of cell types (e.g., cardiomyocytes, fibroblasts). In summary, these findings suggest an increasing trend in the number of molecular players involved in the pathogenesis of AF as the disease progresses.

**TABLE 4:** The number of differentially expressed genes and pathways in the atrial appendage tissues and cells of goat hearts during the transition period and chronic period of AF.

Type	Sample_category	T-S (Up/Down)	C-S (Up/Down)
Gene	LAA	1140 (654/486)	1782 (1011/771)
	RAA	1104 (507/597)	841 (482/359)
	cmLAA	1160 (484/676)	1259 (537/722)
	cmRAA	579 (287/292)	1072 (483/589)
Pathway	LAA	1010 (564/446)	1997 (1168/829)
	RAA	758 (305/453)	539 (329/210)
	cmLAA	1004 (302/702)	1276 (489/787)
	cmRAA	703 (317/386)	1211 (444/767)

AF, atrial fibrillation; cmLAA, left atrial appendage for cardiac muscle cell; cmRAA, right atrial appendage for cardiac muscle cell; C-S, Chronic-Sham; LAA, left atrial appendage for cardiac muscle tissue; RAA, right atrial appendage for cardiac muscle tissue; T-S, Transition-Sham.

**FIGURE 6:**
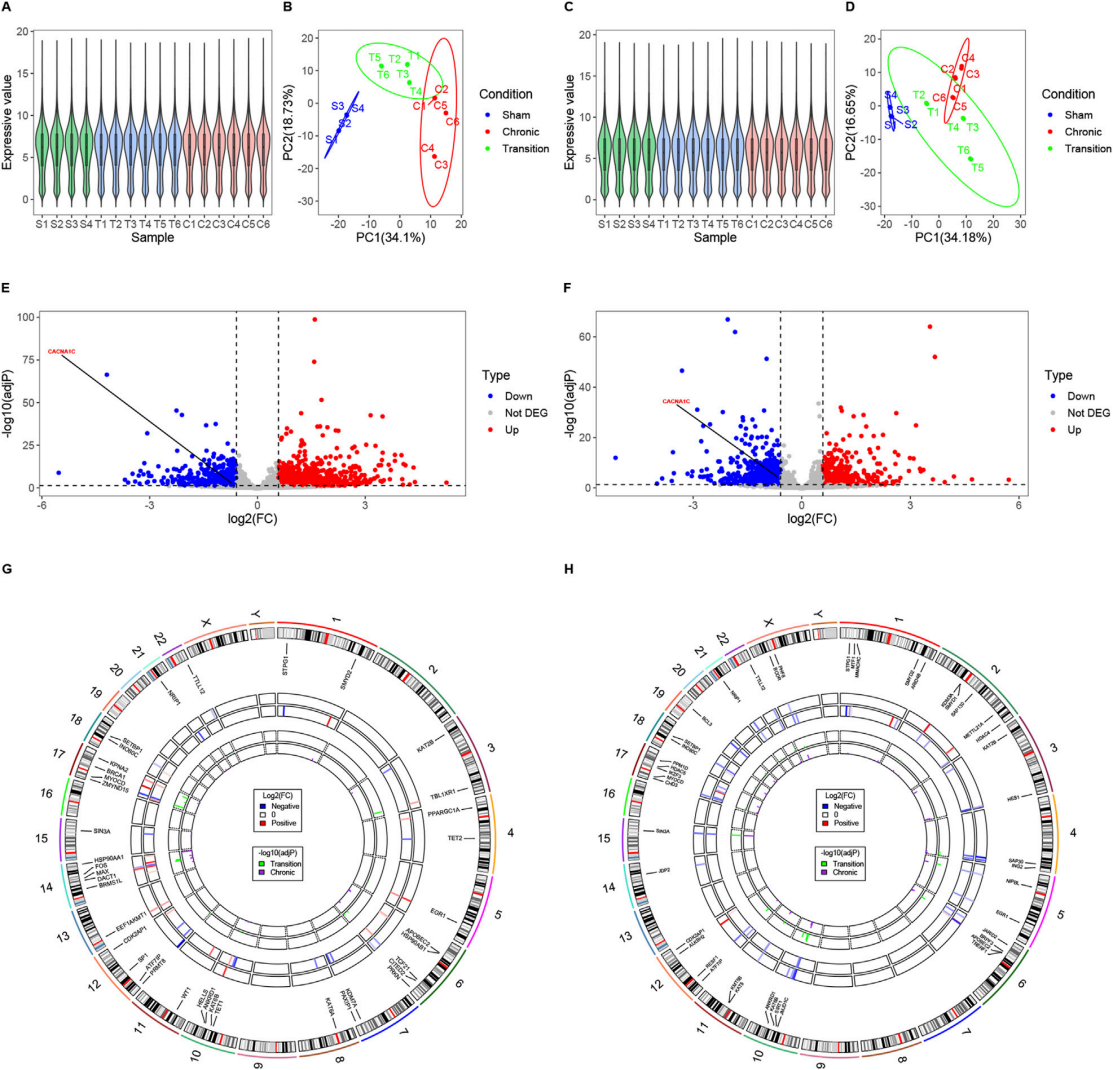
Examining potential epigenetic regulatory genes associated with the CACNA1C gene in LAA and cmLAA. **(A)** Violin plots of normalized samples from the three groups in LAA. S, T, and C on the *x*-axis represent the Sham group, Transition group, and Chronic group, respectively. **(B)** Principal component analysis plot of the three groups in LAA. **(C)** Violin plots of normalized samples from the three groups in cmLAA. **(D)** Principal component analysis plot of the three groups in cmLAA. **(E)** Volcano plot showing differentially expressed genes in the Chronic group of LAA. Red dots represent upregulated genes, while blue dots represent downregulated genes. **(F)** *Volcano* plot showing differentially expressed genes in the Chronic group of cmLAA. **(G)** Log2(FC) changes and -log10 (adj*P*) values of differentially expressed EpiGeneSet genes in the Transition and Chronic groups of LAA. The circle plot has seven tracks from the outer to the inner. Track 1: Chromosome scale; Track 2: Chromosome band staining; Track 3: Labels of EpiGeneSet genes with intergroup differences; Track 4: Log2(FC) changes between Sham and Transition groups, with red gradients indicating upregulation and blue gradients indicating downregulation; Track 5: Log2(FC) changes between Sham and Chronic groups; Tracks 6 and 7: log10 (adj*P*) values for the Sham-Transition and Sham-Chronic comparisons, respectively. **(H)** Log2(FC) changes and -log10 (adjP) values of differentially expressed EpiGeneSet genes in the Transition and Chronic groups of cmLAA. Data were derived from RNA-sequencing of sheep AF model using left atrial appendage rapid pacing. cmLAA: left atrial appendage for cardiac muscle cell; LAA: left atrial appendage for cardiac muscle tissue.

Normalization and PCA of samples from LAA and cmLAA demonstrate uniform and distinct clustering within groups, as shown in Figures 6A–D. Volcano plots illustrate the downregulation of CACNA1C expression in both LAA and cmLAA during the Chronic phase (adj*P* < 0.05), as depicted in Figures 6E, F. However, during the Transition phase, the expression of CACNA1C either remains unchanged or exhibits inconsistent expression in LAA and cmLAA (not visualized). Intersection of the EpiGeneSet gene set with the DEG sets from these two sample types was visualized using circle diagram, displaying fold changes and adj*P* values for Transition and Chronic groups, as seen in Figures 6G, H. By combining DEGs from LAA and cmLAA, we identified 2 genes in the Transition group and 11 genes in the Chronic group, including ATF7IP and KAT2B. Additionally, for RAA and cmRAA, normalization, PCA, and circle diagrams are presented in Supplementary Figure S1A–F. Combining with the EpiGeneSet gene set, we identified 2 DEGs in the Transition group and 5 DEGs in the Chronic group. In summary, DEGs related to DNA methylation, filtered based on the EpiGeneSet gene set, are presented in Supplementary Figure S2 for both Transition and Chronic groups. Notably, despite differences in grouping and analytical methods compared to the original literature, the overall DEGs obtained from our analysis were largely similar.

### 3.7 Epigenetic regulatory pathways potentially related to the CACNA1C gene were identified using RNA-sequencing

The GSVA analysis results for the above eight DEG sets are presented in Supplementary Table S6, while the last four rows of Table 4 summarize the number of differentially expressed pathways between each group (Transition-Sham or Chronic-Sham) for the eight datasets. These results suggest that the distribution trend of differential pathways in the left and right atrial appendages and different AF progression states is similar to the numeric trend of DEGs, with more left and less right atrial appendages and more in the chronic phase and less in the transition phase.

**FIGURE 7:**
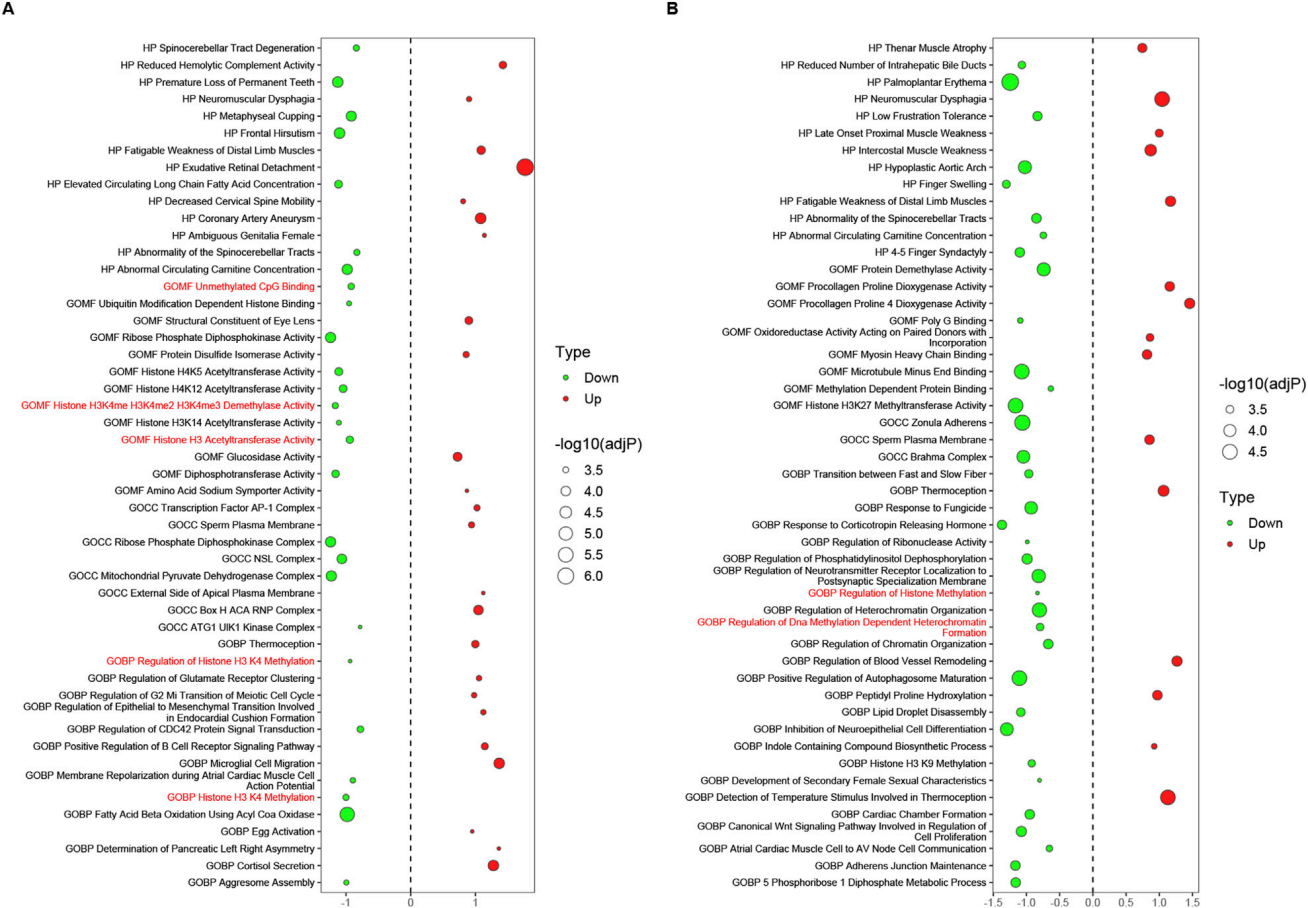
Epigenetic regulatory pathways potentially related to the CACNA1C gene during the chronic phase, observed in LAA and cmLAA. **(A)** Bubble plot of all pathways in EpiPathway50 for LAA. The bubble size represents -log10 (adjP), where red bubbles indicate upregulated pathways, and green bubbles indicate downregulated pathways. The size of the bubbles reflects the intensity of differences between groups, with red-labeled pathways on the *Y*-axis signifying potential epigenetic regulatory pathways associated with the CACNA1C gene. **(B)** Bubble plot of all pathways in EpiPathway50 for cmLAA. cmLAA: left atrial appendage for cardiac muscle cell; LAA: left atrial appendage for cardiac muscle tissue.

The datasets in Supplementary Table S6 were screened and reconstructed as follows: the first four datasets composed of tissues were pairwise combined with their corresponding cell datasets, such as LAA_TvS with cmLAA_TvS. Then, these two datasets were recombined into new datasets based on their shared pathways (sorted by adj*P* in ascending order), and the top 50 pathways were selected to form the final datasets EpiPathway50_LAA_TvS and EpiPathway50_cmLAA_TvS. A total of four pairs of combinations were obtained, as summarized in Supplementary Table S7. The results showed that no epigenetic regulatory pathways were found in all four Transition phases, but some functional pathways related to cardiac electrophysiology, such as Channel Activator Activity, were observed. In EpiPathway50_LAA_CvS (left atrial appendage tissue during the chronic phase) and EpiPathway50_cmLAA_CvS, seven potential epigenetic regulatory pathways were identified, such as Unmethylated CpG Binding. Additionally, some pathways related to cardiac matrix remodeling, such as Regulation of Blood Vessel Remodeling, were observed and visualized in Figures 7A, B. Furthermore, we found six potential epigenetic regulatory pathways, such as Histone H3 Acetyltransferase Activity, in EpiPathway50_RAA_CvS and EpiPathway50_cmRAA_CvS, which are visualized in Supplementary Figures S3A, B. By merging overlapping pathways, we obtained a final set of eight differential epigenetic pathways, whose functional changes mainly involve DNA methylation, H3K27 acetylation, and H3K4me1, all showing a downregulation trend. These pathways are likely related to the maintenance of the DNA methylation status of the CACNA1C gene. Although the mining methods used in this study differ from those in the original literature, many of the pathways mentioned in the original literature were replicated. In summary, the data indicate that the changes in DNA methylation pathway related to AF mainly occurs during the chronic phase and involves both the left and right atrial appendages.

### 3.8 ATF7IP and KAT2B genes identified may regulate DNA methylation of the CACNA1C gene

We merged all genes from the aforementioned Eight differentially expressed epigenetic pathways based on identical gene names, resulting in a gene set named EpiPathwaySet containing 72 genes, as shown in Sheet 1 of Supplementary Table S8. Four datasets related to the chronic phase in Supplementary Table S5 were reconstructed using these 72 genes, and the Pearson correlation coefficients between their expression values and the expression of the CACNA1C gene were calculated. The top 30 genes with the strongest correlations, sorted from strongest to weakest, were used to create a network plot. Genes with co-expression characteristics with the CACNA1C gene were identified using a cutoff value of |R| > 0.68 and *p* < 0.05, as shown in Sheet 2–5 of Supplementary Table S8.

**FIGURE 8:**
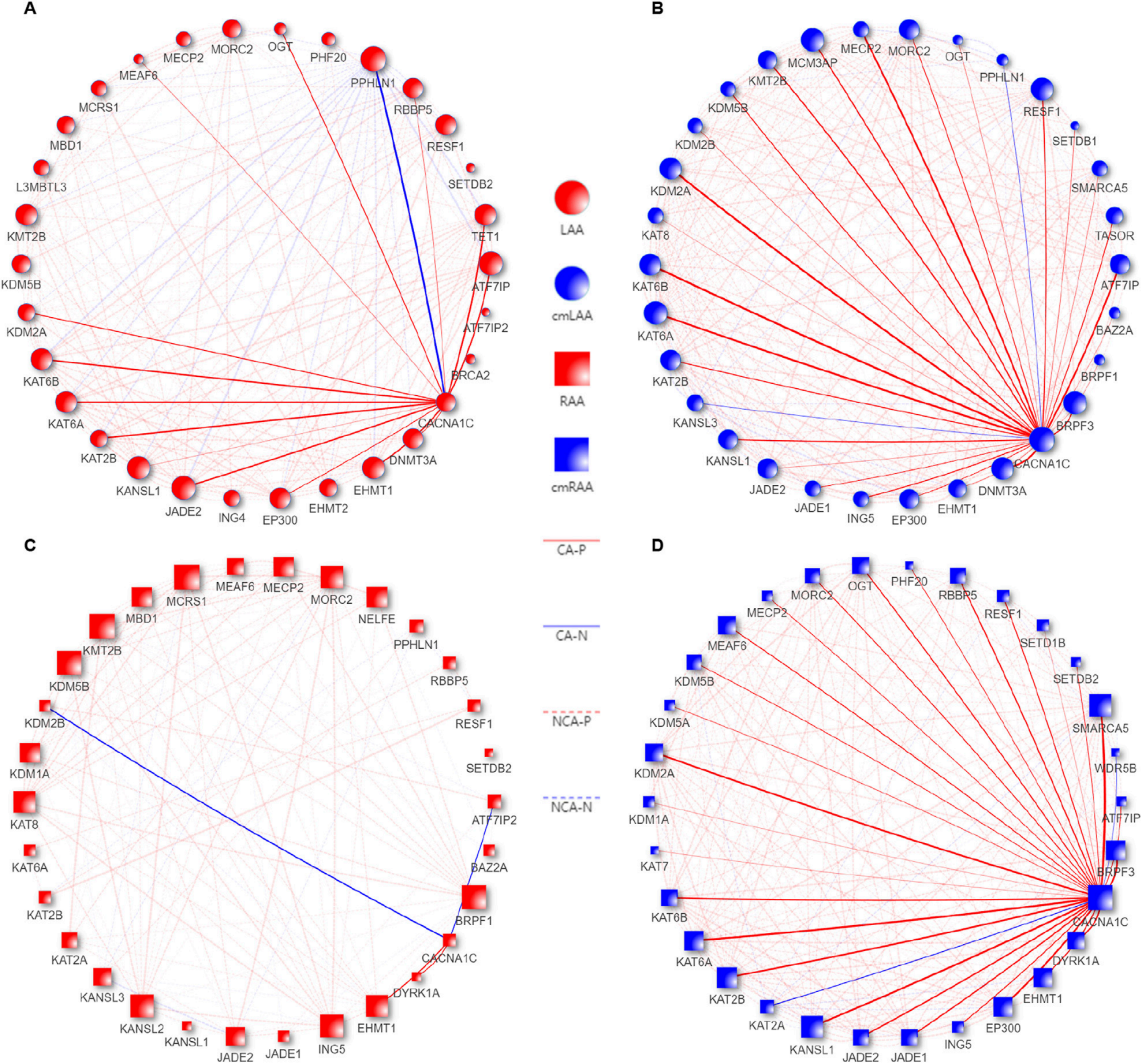
Co-expression pattern diagrams of EpiPathwaySet genes and the CACNA1C gene in the Chronic groups of LAA, RAA, cmLAA, and cmRAA. **(A)** Co-expression pattern diagram of EpiPathwaySet genes and the CACNA1C gene in LAA. **(B)** Co-expression pattern diagram of EpiPathwaySet genes and the CACNA1C gene in cmLAA. **(C)** Co-expression pattern diagram of EpiPathwaySet genes and the CACNA1C gene in RAA. **(D)** Co-expression pattern diagram of EpiPathwaySet genes and the CACNA1C gene in cmRAA. The size of the nodes represents the number of edges connected to them, with red nodes representing myocardial tissue, blue nodes representing cardiomyocytes, circular nodes representing the left atrial appendage, and square nodes representing the right atrial appendage. CA-N: Solid blue lines represent the degree of negative correlation between EpiPathwaySet genes and the CACNA1C gene; CA-P: Solid red lines represent the degree of positive correlation between EpiPathwaySet genes and the CACNA1C gene; cmLAA: left atrial appendage for cardiac muscle cell; cmRAA: right atrial appendage for cardiac muscle cell; NCA-N: Dashed blue progressive lines represent the degree of negative correlation between EpiPathwaySet genes; NCA-P: Dashed red progressive lines represent the degree of positive correlation between EpiPathwaySet genes; LAA: left atrial appendage for cardiac muscle tissue; RAA: right atrial appendage for cardiac muscle tissue.

As we observed in Figures 8A, B, the gene set LAA_cmLAA_overlapping, which is related to CACNA1C and overlaps in LAA and cmLAA, includes ATF7IP, KAT2B, DNMT3A, and others. In Figures 8C, D (RAA and cmRAA), we observed another overlapping gene set, RAA_cmRAA_overlapping, as detailed in Sheet 6, 7 of Supplementary Table S8. Finally, we intersected these overlapping genes with the DEGs corresponding to the chronic phase of AF in Supplementary Figure S2, and determined that ATF7IP and KAT2B are likely involved in the epigenetic regulation of the CACNA1C gene in left atrial appendage.

## 4 Discussion

Studies linking DNA methylation of the CACNA1C gene to AF are relatively rare, especially those integrating multi-omics approaches. In this study, HepG2 cells treated with AZA showed increased CACNA1C expression, and this result was further validated in T47D estrogen-resistant cell lines. Analysis of DNA methylation array data revealed significant differences in methylation at a CpG site in intron 30 of the CACNA1C gene between AF and SR groups. Pyrosequencing was used to assess methylation at three CpG sites in this region from human left atrial appendage tissue, confirming their association with AF. Based on ChIP-sequencing data, we observed that this region is enriched for enhancer markers such as CTCF, H3K27ac, and H3K4me1, suggesting that methylation changes in this region could influence CACNA1C gene expression. Using functional annotation data from the MSigDB database, we customized an EpiGeneSet containing genes related to epigenetic regulation. Subsequent analysis with GVSA helped define the EpiPathwaySet, which, together with CACNA1C co-expression patterns and methylation data, suggested that ATF7IP and KAT2B may participate in the epigenetic regulation of intron 30 of CACNA1C. In this study, the selection criteria and process for the cell lines and datasets used are detailed in Supplementary File S1.

AZA is an adenosine analog of natural 2′-deoxycytidine, which reduces DNA methylation by inhibiting DNA methyltransferase. It is used clinically to treat leukemia, as it prevents tumor cell proliferation and drug resistance, targeting the S-phase of the cell cycle (Jabbour and Kantarjian, 2022). In research, it is widely employed as a tool in DNA methylation studies. Animal studies have shown that AZA can reverse hypermethylation in the Pitx2c promoter region in a rat heart failure model, alleviating heart failure symptoms (Kao et al., 2013). Additionally, long-term administration of AZA has been shown to improve ECG arrhythmia and reduce left ventricular fibrosis in a rat model of spontaneous hypertension (Donate Puertas et al., 2017). In this study, AZA treatment in HepG2 and T47D cells significantly increased CACNA1C expression, indicating that AZA may regulate CACNA1C through the DNA methylation pathway, offering insights for the development of epigenetic drugs related to AF.

The human CACNA1C gene is located on chromosome 12p13 and has multiple transcription start sites and transcripts. Among these, transcript 15 is expressed most abundantly in cardiomyocytes, consisting of 47 exons and encoding a protein of 2,138 amino acids. Studies on epigenetic modifications related to AF have shown that the regulation of CACNA1C expression is primarily associated with microRNA interference (Binas et al., 2020), while research on DNA methylation remains scarce. A study by Zhao et al. using chip data identified a CpG site in intron 30 of the CACNA1C gene as being associated with AF, though further validation was not performed (Zhao et al., 2017). In our study, we reanalyzed the chip data and discovered two additional CpG sites near the previously identified one. Further analysis showed that the methylation levels of these three sites exhibited collinearity, and their association with AF was confirmed in human left atrial appendage tissue. Moreover, the Framingham Heart Study previously reported several DNA methylation sites associated with AF (Lin et al., 2017), but these did not overlap with the sites identified in our study, potentially due to differences in sample types (blood vs. tissue) and population heterogeneity (Caucasian vs. Asian).

Through ChIP-sequencing in MCF-7 and other cell lines, we identified strong enhancer signals, including H3K27ac, H3K4me1, and CTCF, enriched in this region. The ROSE software predicted the presence of a TE at this location. Third-party datasets confirmed the association between this enhancer’s activity and CACNA1C expression. We hypothesize that this TE may similarly regulate CACNA1C expression in AF, influenced by the three identified CpG sites. Studies have shown that TE are special regulatory regions in the genome that can significantly activate gene expression. They typically enrich histone modification markers such as H3K27ac and H3K4me1, which are commonly used to identify active enhancer regions, while H3K4me3 primarily marks active promoter regions. Additionally, CTCF protein helps enhancers interact precisely with target genes by regulating chromatin structure and forming topological domain boundaries. Furthermore, H3K27ac and H3K4me1 can promote recruitment of various transcription factors in enhancer regions, thereby initiating the formation of macromolecular complexes—SE—which regulate downstream genes in a condensed state, while CTCF protein, as a regulatory factor, also participates in the formation and maintenance of SE (Kravchuk et al., 2023). Although this site in MCF-7 cells was only classified as a TE, further research is needed to determine whether it functions as a super-enhancer in AF, requiring additional biological exploration and experimental validation.

In this study, we identified ATF7IP and KAT2B as potential contributors to the epigenetic regulation of the CACNA1C gene within intron 30 by analyzing differentially expressed epigenetic genes and pathways in conjunction with their Pearson correlation coefficients with CACNA1C. Earlier research found that the ATF7IP gene (also known as MCAF1) functions as a partner gene of the histone methyltransferase SETDB1, stabilizing H3K9me3 and thereby promoting heterochromatin maintenance or gene repression (Timms et al., 2016). Recent studies suggest that ATF7IP may inhibit cardiac reprogramming initiated by transcription factors like Mef2c, Gata4, Tbx5, and Hand2, which convert cardiac fibroblasts into induced cardiomyocytes (iCMs). Knockdown of ATF7IP in this context was shown to improve acute myocardial infarction outcomes (Missinato et al., 2023). We hypothesize that the downregulation of ATF7IP during the chronic phase of AF may act as a cardioprotective mechanism. The gene KAT2B (also known as PCAF) encodes a protein that interacts with p300/CBP and belongs to the GCN-5-related N-acetyltransferase (GNAT) superfamily. It exhibits significant histone H3 acetylation activity and is involved in apoptosis, inflammation, and oxidative stress (Jin et al., 2011). One study found that downregulation of KAT2B reduces cardiomyocyte apoptosis by inhibiting the NF-κB pathway, thus protecting against myocardial ischemia-reperfusion injury (MIRI) (Koutelou et al., 2021). Another study linked KAT2B variants rs3021408 and rs17006625 with an increased risk of coronary artery disease in the Han Chinese population (Hou et al., 2020). These genes are known to be associated with DNA methylation, and in our study, ATF7IP and KAT2B exhibited strong co-expression with CACNA1C in both LAA and cmLAA tissues, suggesting a significant epigenetic regulatory role. The specific mechanisms through which these genes influence DNA methylation at the three CpG sites of CACNA1C require further investigation. Nonetheless, the identification of these DNA methylation biomarkers in the AF model may provide insights for future drug development. Additionally, it is noteworthy that while DNMT3A expression did not meet the defined threshold, it was close and showed a strong similarity to CACNA1C expression, suggesting that a reduction in this enzyme may directly lead to decreased DNA methylation at the three CpG sites of the CACNA1C gene.

**FIGURE 9:**
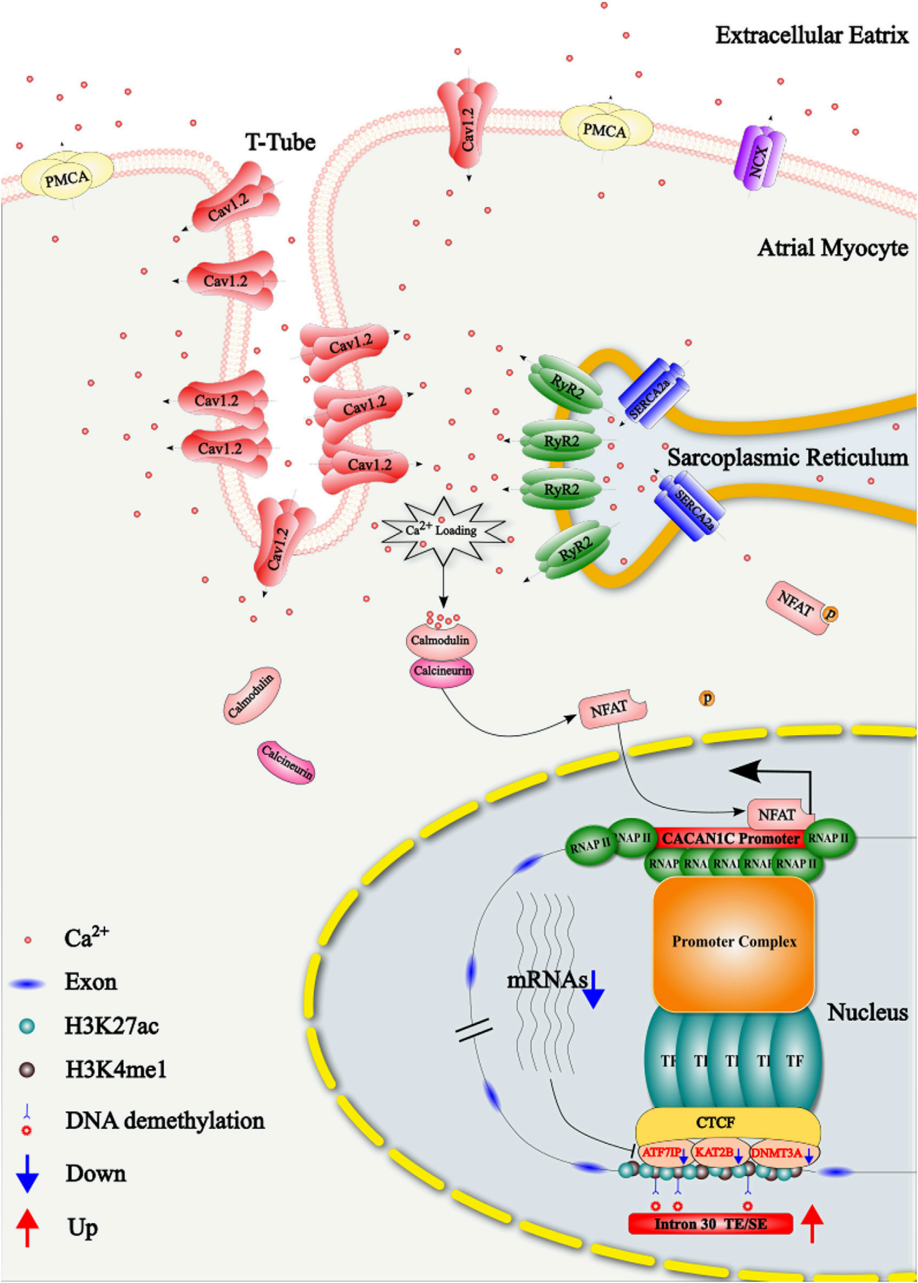
Schematic representation of the regulatory mechanism by which the DNA methylation of three CpG sites in intron 30 of the CACNA1C gene influences the reduction of Cav1.2 protein during atrial fibrillation (AF). TE: Typical Enhancer; SE: Super Enhancer.

Additionally, in the left atrial appendage tissue and cardiomyocytes of sheep AF model, the expression of the CACNA1C gene was reduced in the Chronic group compared to the Sham group. Based on other findings from this study, we propose the following mechanism: prolonged AF leads to calcium overload in cardiomyocytes, resulting in increased calcium binding to calmodulin and recruitment of calcineurin. This complex promotes dephosphorylation of cytoplasmic NFAT, which subsequently translocates to the nucleus and binds to the promoter region of CACNA1C, reducing its expression (Nattel et al., 2020). The prolonged reduction in the expression of this gene may result in a feedback mechanism that also decreases the regulatory genes ATF7IP, KAT2B, and DNMT3A. This, in turn, leads to DNA demethylation at the three CpG sites in the region, which increases the activity of the TE/SE elements at this locus. Consequently, this boosts the relative expression of the gene, although its overall expression remains lower. The mechanism is illustrated in Figure 9. This regulatory pathway might play a limited role in reversing the downregulation of CACNA1C during AF. However, targeted pharmacological interventions that enhance this reversal effect could provide a therapeutic approach to mitigate the persistent state of AF. In summary, CACNA1C is a critical housekeeping gene subject to epigenetic regulation, as well as other factors like transcription factors, long non-coding RNAs, drugs, hormones, and disease states (Jiang and Zhang, 2023). While no previous studies have investigated the regulatory role of this region as a TE or SE in AF, our limited data suggest this possibility, but further experimental validation and larger datasets are needed. Moreover, other genomic approaches (e.g., single-cell sequencing, ATAC-seq), proteomics, and metabolomics were not addressed in this study due to the current scarcity of AF-related data in public databases.

## 5 Conclusion

Based on multi-omics analysis, this study identified that DNA methylation changes at three CpG sites in intron 30 of the CACNA1C gene are associated with AF, and potentially regulated by ATF7IP and KAT2B genes. These findings suggest that the epigenetic markers may provide possible targets for AF diagnosis and drug therapy, and serve as valuable resources to guide future epigenetic-specific experiments related to AF mechanisms.

## Data Availability

The raw omic datasets was deposited in the Gene Expression Omnibus database (GEO) of NCBI. Accession numbers: GSE138255, GSE153557, GSE154681, GSE158081, GSE179049, GSE180652, GSE185259, GSE210475, GSE220254, GSE235681, GSE240902, GSE246499, GSE261518, GSE262470, GSE269415, GSE62727, GSE78113, GSE85353, GSE85536.

## Supplementary material

SUPPLEMENTARY FIGURE S1 Examining potential epigenetic regulatory genes associated with the CACNA1C gene in RAA and cmRAA. **(A)** Violin plots of normalized samples from the three groups in RAA. S, T, and C on the *x*-axis represent the Sham group, Transition group, and Chronic group, respectively. **(B)** Principal component analysis plot of the three groups in RAA. **(C)** Violin plots of normalized samples from the three groups in cmRAA. **(D)** Principal component analysis plot of the three groups in cmRAA. **(E)** Volcano plot showing differentially expressed genes in the Chronic group of RAA. Red dots represent upregulated genes, while blue dots represent downregulated genes. **(F)** *Volcano* plot showing differentially expressed genes in the Chronic group of cmRAA. **(G)** Log2(FC) changes and -log10(adj*P*) values of differentially expressed EpiGeneSet genes in the Transition and Chronic groups of RAA. The circle plot has seven tracks from the outer to the inner. Track 1: Chromosome scale; Track 2: Chromosome band staining; Track 3: Labels of EpiGeneSet genes with intergroup differences; Track 4: Log2(FC) changes between Sham and Transition groups, with red gradients indicating upregulation and blue gradients indicating downregulation; Track 5: Log2(FC) changes between Sham and Chronic groups; Tracks 6 and 7: log10(adj*P*) values for the Sham-Transition and Sham-Chronic comparisons, respectively. **(H)** Log2(FC) changes and -log10(adjP) values of differentially expressed EpiGeneSet genes in the Transition and Chronic groups of cmRAA. Data were derived from RNA-sequencing of goat AF model using left atrial appendage rapid pacing. cmRAA: right atrial appendage for cardiac muscle cell; RAA: right atrial appendage for cardiac muscle tissue.

SUPPLEMENTARY FIGURE S2 Wordcloud diagram of differentially expressed genes related to DNA methylation screened based on the EpiGeneSet gene set in the Transition or Chronic groups. **(A)** Final differential genes after merging the Transition groups of LAA and cmLAA. **(B)** Final differential genes after merging the Chronic groups of LAA and cmLAA. **(C)** Final differential genes after merging the Transition groups of RAA and cmRAA. **(D)** Final differential genes after merging the Chronic groups of RAA and cmRAA. Red represents upregulated genes, blue represents downregulated genes, and their size represents the enrichment value of expression differences. cmLAA: left atrial appendage for cardiac muscle cell; cmRAA: right atrial appendage for cardiac muscle cell; LAA: left atrial appendage for cardiac muscle tissue; RAA: right atrial appendage for cardiac muscle tissue.

SUPPLEMENTARY FIGURE S3 Epigenetic regulatory pathways potentially related to the CACNA1C gene during the chronic phase, observed in RAA and cmRAA. **(A)** Bubble plot of all pathways in EpiPathway50 for RAA. The bubble size represents -log10(adjP), where red bubbles indicate upregulated pathways, and green bubbles indicate downregulated pathways. The size of the bubbles reflects the intensity of differences between groups, with red-labeled pathways on the *Y*-axis signifying potential epigenetic regulatory pathways associated with the CACNA1C gene. **(B)** Bubble plot of all pathways in EpiPathway50 for cmRAA. cmRAA: right atrial appendage for cardiac muscle cell; RAA: right atrial appendage for cardiac muscle tissue.

SUPPLEMENTARY TABLE S5 Differentially expressed genes from DESeq2.

SUPPLEMENTARY TABLE S6 Differentially expressed pathways from GSVA.

SUPPLEMENTARY TABLE S7 EpiPathway50.

SUPPLEMENTARY TABLE S8 The results of KEGG analysis for the blue module.
