# Supplementary figures and images for: The combination of decitabine with multi-omics confirms the regulatory pattern of the correlation between DNA methylation of the CACNA1C gene and atrial fibrillation
Source: Front Pharmacol. 2024 Dec 13;15:1497977. doi: 10.3389/fphar.2024.1497977 (PMC11681619; doi:10.3389/fphar.2024.1497977)

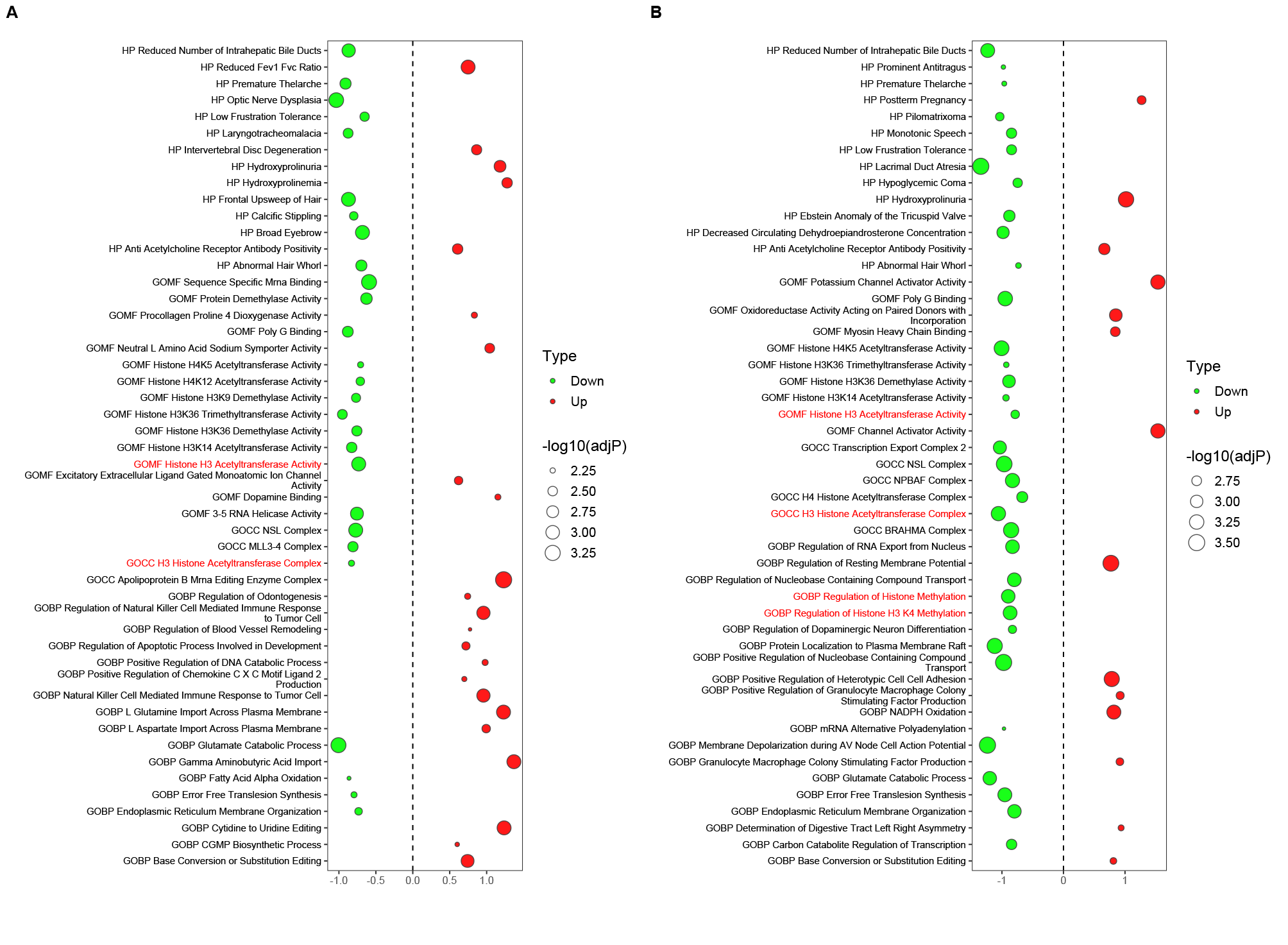

Supplement: Supplementary file 3 [file Image3.tif]

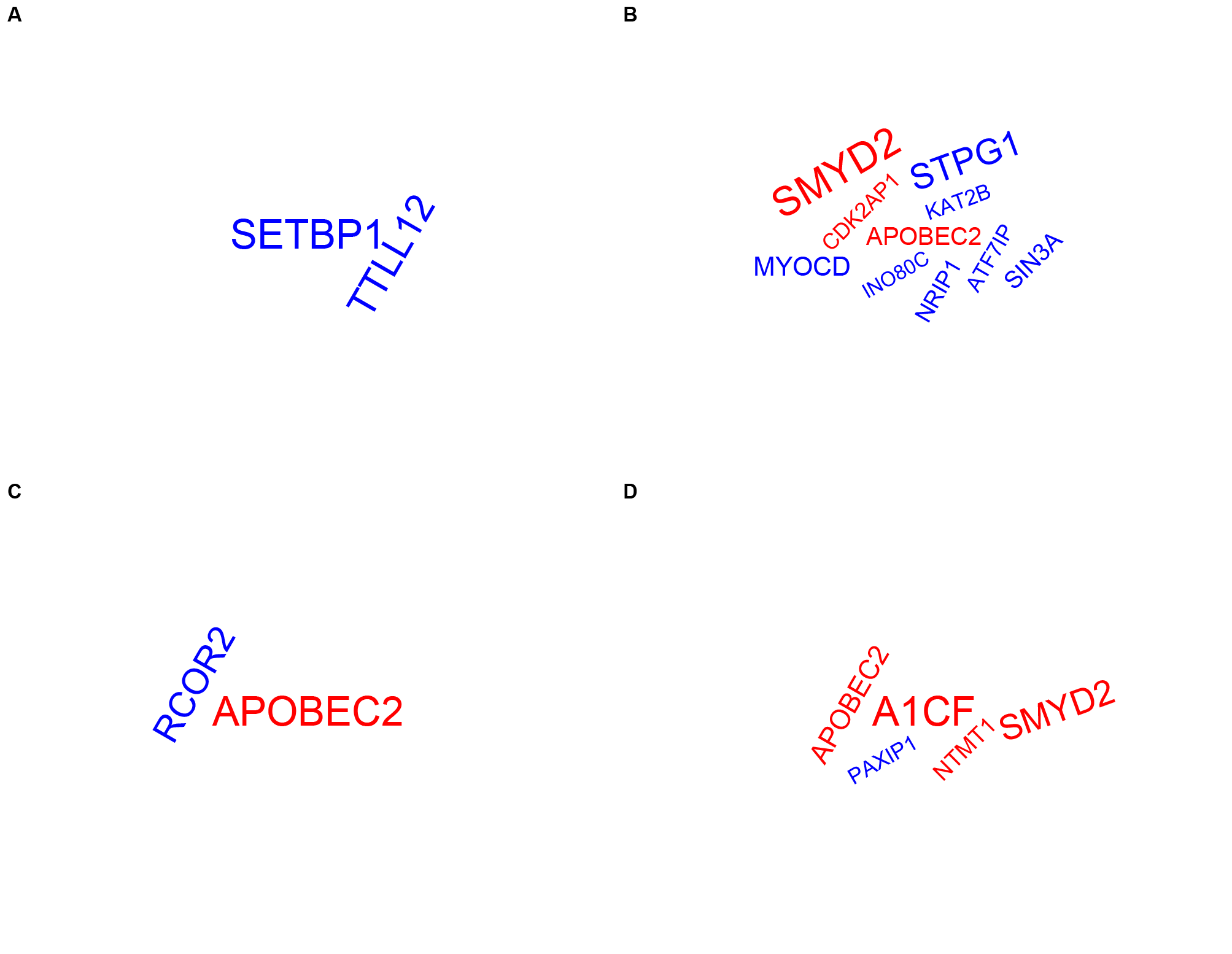

Supplement: Supplementary file 4 [file Image2.tif]

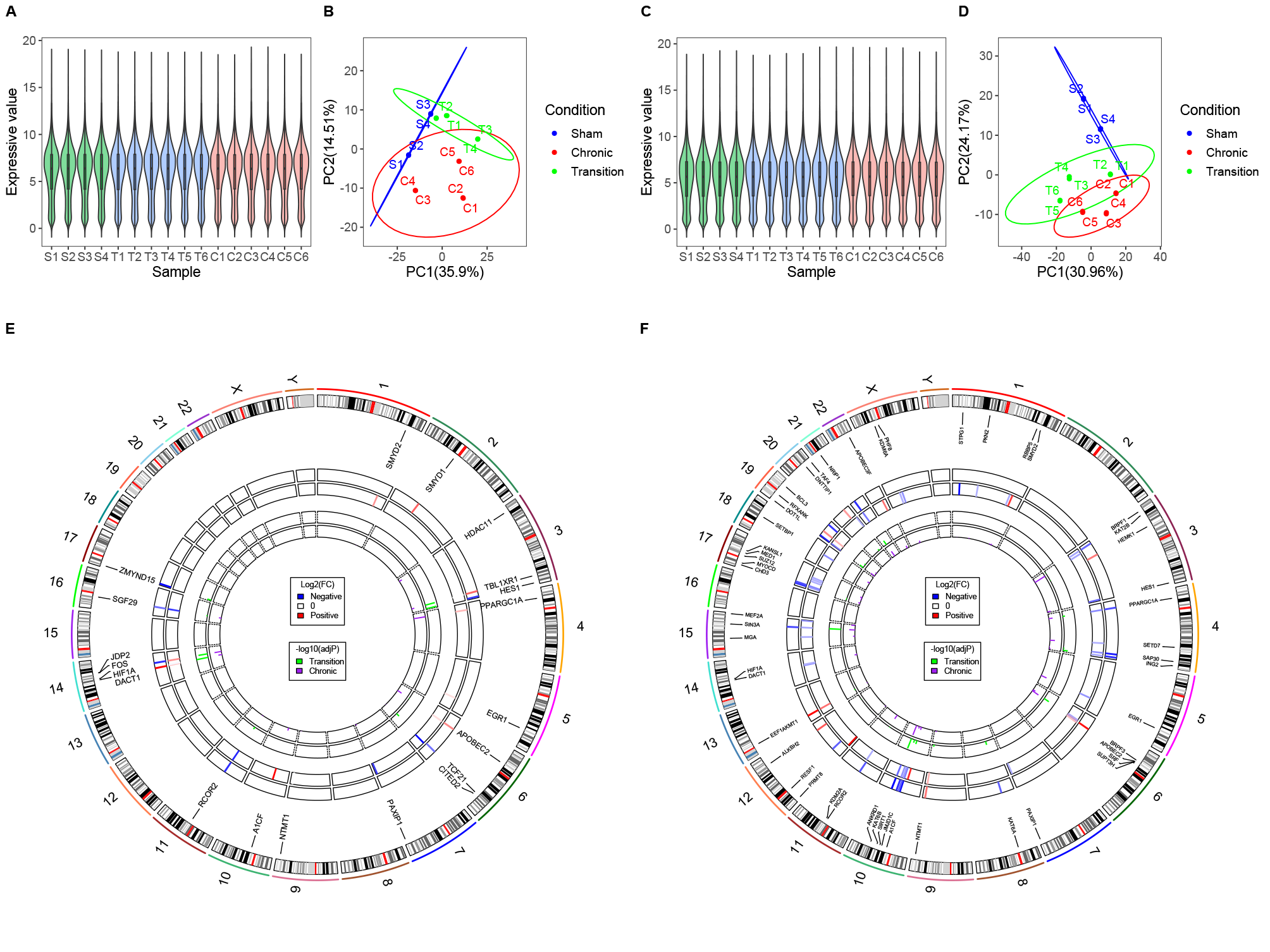

Supplement: Supplementary file 5 [file Image1.tif]
