# Supplementary material for: The combination of decitabine with multi-omics confirms the regulatory pattern of the correlation between DNA methylation of the CACNA1C gene and atrial fibrillation
Source: Front Pharmacol. 2024 Dec 13;15:1497977. doi: 10.3389/fphar.2024.1497977 (PMC11681619; doi:10.3389/fphar.2024.1497977)
Supplement: Supplementary file 12 [file DataSheet1.docx]

**The selection criteria and process for the cell lines and datasets used in this study**

**Selection of HepG2 Cell Line**

The initial scientific concept for this study was inspired by another research direction in our group, specifically the observation mentioned in the introduction that 'our previous treatment of HepG2 with AZA showed that CYP3A4 gene is affected by DNA methylation (PMID: 33048369).' This was one of the reasons we chose HepG2 cells to observe whether AZA affects CACNA1C gene expression. Additionally, through literature review, we found many studies showing this cell line is regulated by DNA methylation, such as PMID: 21295543, PMID: 22939515, PMID: 27081315, PMID: 32068100, etc. Therefore, this cell line was included in our study.

**Selection of Expression Microarray Dataset GSE85536**

This dataset was selected based on the results from HepG2 cell experiments, which showed that AZA could affect CACNA1C expression. To find similar replications in other research results, we searched the GEO database using the terms: (("dna methylation"[MeSH Terms] OR DNA methylation[All Fields]) AND Decitabine[All Fields]) AND "Homo sapiens"[porgn] AND "Expression profiling by array"[Filter]. We excluded datasets with fewer than 3 sample replicates within groups and datasets using Decitabine in combination with other drugs, obtaining 5 datasets in total. Using GEO2R software for differential expression analysis, with thresholds of adj*P* < 0.05 and |log2FC| > 0.58, we preliminarily determined that CACNA1C expression showed statistical significance in dataset GSE85536, which was included for subsequent analysis.

**Selection of ChIP Datasets**

While observing the positions of three CpG sites in CACNA1C gene's intron 30 using the UCSC Genome Browser, we unexpectedly found extensive enrichment signals of ChIP-seq proteins in the H3K27ac and H3K4me1 tracks. These signal data came from 7 built-in UCSC datasets, corresponding to 7 cell lines (HSMM, HUVEC, NHLF, GM12878, K562, NHEK, and hESC). The final results are shown in **Figure 4A**.

Since we couldn't find corresponding expression data for these cell lines in the same datasets, we searched for expression datasets for all 7 cell lines in the GEO database. The search terms were: X[All Fields] AND "Expression profiling by high throughput sequencing"[Filter], where X represents the names of the 7 cell lines, generating 7 search result sets. To narrow down the results, we added filtering attributes and used GEO2R software to calculate TPM values for the CACNA1C gene to determine its expression. We set the common threshold of 0.1 TPM - below this value indicates no expression, above indicates expression. Since each of the 7 search terms still yielded many datasets, we established inclusion criteria for the final dataset selection. They are as follows:

**For Cell Lines without CACNA1C Expression (GM12878, K562, NHEK, and hESC)**

We randomly selected 3 datasets from each search result as input for GEO2R software. If all calculated TPM values for CACNA1C were below 0.1, we considered the gene not expressed in that dataset, included it in the study, and stopped screening. The final results correspond to the top sub-panel in **Figure 4B** of the original manuscript.

**For Cell Lines with CACNA1C Expression (HSMM, HUVEC, and NHLF)**

We randomly selected 3 datasets from each search result as input for GEO2R software. After calculation, if at least one dataset showed a TPM value above 0.1, we considered the gene expressed and recorded the count. If the total count was less than 3, we repeated the above steps until reaching 3 datasets showing expression. The final results correspond to the bottom 3 sub-panels in **Figure 4B** of the original manuscript.

**Selection of ChIP Dataset GSE78113**

To further verify that the three CpG sites in intron 30 of the CACNA1C gene might be located in a potential enhancer or super-enhancer region, we found a ChIP enrichment experiment dataset (GSE78113) in the GEO database that included multiple enhancer markers (including H3K27ac, H3K4me1, H3K4me3, and CTCF). This dataset contains ChIP-sequencing results for both normal and hypoxic Breast Cancer Cell Line MCF-7. The search terms were: (ChIP-Seq[All Fields] AND H3K27ac[All Fields] AND H3K4me1[All Fields] AND ("histone H3 trimethyl Lys4"[All Fields] OR H3K4me3[All Fields]) AND CTCF[All Fields]) AND "Homo sapiens"[porgn]. Due to numerous results, we randomly selected dataset GSE78113 for this study. This dataset includes MCF-7 and 786O cell lines, but only MCF-7 had both normal and hypoxic groups, which was beneficial for comparing gene expression differences between groups. A total of 14 samples were included in this study. The final results are shown in **Figure 5A**.

**Selection of RNA-sequencing Datasets GSE85353 and GSE153557**

However, since GSE78113 dataset didn't include differential gene expression data, we used the search terms: (MCF7[All Fields] AND ("hypoxia"[MeSH Terms] OR hypoxia[All Fields])) AND "Homo sapiens"[porgn] AND "Expression profiling by high throughput sequencing"[Filter] to search for corresponding gene expression datasets, yielding 23 results. Due to the large number of datasets retrieved, we randomly selected three datasets (GSE71401, GSE85353, and GSE153557) as input for GEO2R software to observe CACNA1C gene expression differences between hypoxic and normal groups. GSE71401 showed no change in CACNA1C gene expression and was excluded. GSE85353 and GSE153557 were included in this study. The final results are shown in **Figure 5B-C**.

**Selection of RNA-sequencing Dataset GSE138255**

Finally, to obtain further evidence of the relationship between CACNA1C gene expression and DNA methylation in an atrial fibrillation model, we selected the sheep rapid pacing atrial fibrillation model dataset GSE138255 from the GEO database. The search terms were: ("atrial fibrillation"[MeSH Terms] OR atrial fibrillation[All Fields]) AND ("sheep, domestic"[MeSH Terms] OR "sheep"[MeSH Terms] OR "Ovis aries"[Organism] OR sheep[All Fields]) AND "Expression profiling by high throughput sequencing"[Filter]. Compared to small animal models such as mice, rats, and rabbits, the sheep model more closely resembles human cardiac physiological structure, with more controllable external influencing factors.
